# A Comparison of Motor Unit Control Strategies between Two Different Isometric Tasks

**DOI:** 10.3390/ijerph17082799

**Published:** 2020-04-18

**Authors:** Sunggun Jeon, William M. Miller, Xin Ye

**Affiliations:** Department of Health, Exercise Science, and Recreation Management, The University of Mississippi, Oxford, MS 38677, USA; sjeon3@go.olemiss.edu (S.J.); wmmille2@go.olemiss.edu (W.M.M.)

**Keywords:** surface EMG decomposition, motor unit, mean firing rate, recruitment threshold, derecruitment threshold, task differences

## Abstract

Background: This study examined the motor unit (MU) control strategies for non-fatiguing isometric elbow flexion tasks at 40% and 70% maximal voluntary isometric contraction. Methods: Nineteen healthy individuals performed two submaximal tasks with similar torque levels: contracting against an immovable object (force task), and maintaining the elbow joint angle against an external load (position task). Surface electromyographic (EMG) signals were collected from the agonist and antagonist muscles. The signals from the agonist were decomposed into individual action potential trains. The linear regression analysis was used to examine the MU recruitment threshold (RT) versus mean firing rates (MFR), and RT versus derecruitment threshold (DT) relationships. Results: Both agonist and antagonist muscles’ EMG amplitudes did not differ between two tasks. The linear slopes of the MU RT versus MFR and RT versus DT relationships during the position task were more negative (*p* = 0.010) and more positive (*p* = 0.023), respectively, when compared to the force task. Conclusions: To produce a similar force output, the position task may rely less on the recruitment of relatively high-threshold MUs. Additionally, as the force output decreases, MUs tend to derecruit at a higher force level during the position task.

## 1. Introduction

Skeletal muscle contractions consist of dynamic (e.g., concentric and eccentric contractions) and static muscle actions (e.g., isometric contraction). Based on the load type applied on the muscle, the isometric muscle actions can occur during two different tasks: contracting muscles against an immovable object (force task), and holding still against an external load (position task). Interestingly, even with the similar mechanical torque requirements for both isometric tasks, it is well-established that the fatiguing isometric tasks show differences in task failure time [1], myoelectric activities, perceived effort [2,3], and spinal reflex activities [4]. In addition, the variation of fatigue responses in both tasks was also dependent on contraction intensities and limb positions [5,6,7]. Thus, a variety of factors such as the modulations of the motor unit (MU) activities (discharging and recruitment) [8] can contribute to the task-related differences in skeletal muscle fatigue [9].

With the difficulty to identify a single major contributing factor for the task-related differences in muscle fatigue, an alternative way is to study motor control strategies for these tasks directly, during the non-fatiguing condition. For example, by using surface electromyography (EMG) in both sexes, Garner et al. [10] found no difference in the soleus muscle EMG amplitude between the force and position tasks during submaximal plantarflexion across a range of contraction intensities (20–50% of maximal ground reaction force). However, Buchanan and Lloyd [11] reported differential coactivation patterns of EMG amplitude for the synergistic muscles for both non-fatiguing elbow flexion and extension force and position tasks, regardless of sex, suggesting two distinct motor control strategies being used in these tasks. In addition to the global EMG activity, examining MU firing properties may shed more information on understanding the underlying mechanisms of the task-related differences. For example, Mottram et al. [8] examined single MU behavior from biceps brachii in fifteen men using a fine-wire bipolar electrode during the force and position isometric sustained fatiguing contractions at low target force. The results showed that overall MU behaviors were similar regarding MU mean firing rates (MFR) and recruitment threshold (RT) between two tasks. However, the number of recruited MUs was greater during the position task than the force task, suggesting greater excitatory input from the central nervous system (CNS) to motoneuron pool for the position task when compared to the force task [8]. A more recent study compared single MU activity during fatiguing contraction between these two tasks in both sexes, and found that the magnitude of the decline in the MFR was greater in the position task when compared to the force task. In addition, the decline in the RT of detected MUs was present during the position task, but not during the force task. The authors suggest that these changes may involve differential synaptic mechanisms [12]. Thus, despite similar mechanical requirements between two isometric tasks during non-fatiguing contractions, these results from previous studies suggested that two distinct mechanisms of MU control strategies, such as differential MU activities and/or excitatory and inhibitory input to the CNS, may exist in these tasks.

For more than four decades, the development in surface EMG decomposition algorithms has allowed researchers to decompose the surface EMG signals into MU action potential trains [13,14,15,16]. With these techniques, previous studies examined MU activities during submaximal, maximal, or fatiguing contractions using different muscles. Specifically, they found that a negative linear relationship between RT and MFR of the active MUs during a voluntary contraction, which was described as the “onion-skin” scheme [12]. This indicates that the low-threshold MUs tend to have higher firing rates than those of the high-threshold MUs, during the voluntary contractions [5,6,7]. This relationship also suggests an important neural strategy on how the CNS controls the motoneuron pool: the slope of the linear regression line indicates the overall motor control scheme described as the “operating point” of the motoneuron pools [17]. Specifically, when the force output (contraction intensity) increases, the slope and y-intercept of the linear regression line between MU RT and MFR gradually become less negative and higher, respectively, suggesting the recruitment of the additional high-threshold MUs and the increased firing rates of the active MUs to meet the force production demand. On the other hand, the relationship between MU RT and derecruitment threshold (DT) is positive, suggesting that higher threshold MUs generally tend to be derecruited at higher force levels than where they were recruited, but lower threshold MUs tend to be derecruited at lower force level [17,18]. This MU RT vs. DT relationship has been used in a previous study to examine the fatiguing effect on the motor control strategy [19]. Thus, the EMG decomposition algorithm, which examines the active MU action potential trains during voluntary isometric contractions, can serve as a useful tool to investigate MU control mechanisms for non-fatiguing isometric tasks.

Considering previous studies [8,12] have found distinct MU control strategies between the force and position tasks during sustained fatiguing contractions regardless of sex, it is possible to observe two different mechanisms during non-fatiguing force and position tasks. Specifically, the direct comparison of MU control strategies between the non-fatiguing force and position isometric tasks has not been examined, and varying the MU control strategies will potentially influence MU activities and the mechanisms involved. The potential presence of two distinct mechanisms in the two different tasks may provide a better understanding of the underlying mechanism(s) leading to the task differences. However, the knowledge regarding these two tasks per se remains to be poor. Therefore, the primary purpose of this study was to determine the MU control strategies using linear regression analysis during the force and position tasks at 40% and 70% contraction intensities in human biceps brachii muscle. Based on a previous study [20] that reported biceps brachii muscle replying more on the strategy of recruiting MU at high force level (around up to 88%), 40% and 70% contraction intensities were selected to examine MU control strategies between two different isometric tasks. In addition, the surface EMG amplitudes of biceps and triceps brachii were also monitored. Since it has been shown that the EMG amplitude did not differ between two tasks [10], we expected to see a similar finding regarding the EMG amplitude between two tasks. However, considering the different excitatory inputs to the motoneuron pool between two different tasks [21,22], the relationship between MU RT and MFR, as well as the relationship between MU RT and DT between two tasks may differ.

## 2. Materials and Methods

### 2.1. Experimental Design

This study used a within-subject crossover design to examine the MU firing properties of the agonist muscle and the surface EMG amplitude of both the agonist and antagonist muscles during two different types of isometric contraction (force vs. position tasks) at different contraction intensities. All the subjects visited the laboratory three times to complete this investigation. The first visit served as a familiarization session. The second and third visits involved performing two different isometric tasks, which were conducted in a randomized order. At least 48 h of rest was provided between visits. All subjects were right-handed, and they performed the exercise tests with the dominant arm (based on their throwing preferences). The data of this experiment were collected in part with a previously published study [23].

### 2.2. Subjects

Nineteen healthy men (n = 12, mean ± SD: age = 23.7 ± 3.9 years, height = 172.8 ± 5.7 cm, weight = 84.8 ± 12.1 kg) and women (n = 7, mean ± SD: age = 22.3 ± 3.7 years, height = 165.0 ± 2.8 cm, weight = 76.1 ± 12.6 kg) participated in this study. Based on the Physical Activity Guidelines of the U.S. Department of Health and Human Services [24], all subjects were considered physically active (perform at least 150- to 300-min moderate intensity, or 75- to 150-min vigorous intensity, or equivalent combination of aerobic activity once a week with muscle-strengthening activities two or three days a week). The University Institutional Review Board (protocol number: 18-022) approved the experimental procedures. All experimental procedures were in conformity with the policy statement regarding the use of human subjects by the Helsinki Declaration of 1975, as revised in 2008. All subjects completed an informed consent form and a health and exercise status questionnaire before any measurement and investigation. None of them reported neuromuscular, musculoskeletal, or cardiovascular diseases or disorders within six months prior to the investigation. During the consenting process, the subjects were asked to keep their normal activity regarding exercise, food intake, and sleep during the entire investigation. The extra effort was made to conduct testing at roughly the same time of the day.

### 2.3. Procedures

All subjects were introduced to the procedures of the study, and they practiced the exercise tests during the familiarization visit (Visit 1). Specifically, the maximal isometric strength test, the submaximal isometric trapezoid contraction, as well as the two different submaximal isometric contractions (force and position tasks) were practiced. During submaximal trapezoid contractions, we provided visual feedback for the target force of 40% and 70% MVIC to allow participants to become familiarized with the tests. At least 48 h after the familiarization visit, the subjects returned to the laboratory, and they performed one of the randomly assigned exercise visits (Force-Task Visit or Position-Task Visit). Upon arrival, the subjects were seated in front of a custom-designed preacher curl platform with the dominant elbow placed upon the foam cushion on the platform. The platform has a 45-degree angle to the floor. With this posture and the elbow angle position of 135° using a v-shaped steel hinge, the research staff then conducted maximal voluntary isometric contraction (MVIC) tests on both the subjects’ elbow extensors and flexors [23]. Briefly, with the neutral hand position, the subjects were instructed to press the wrist down to an immovable wooden stand, which was firmly supporting the dominant wrist. After several submaximal warmup contractions, the subjects performed 3 sets of 3-s maximal elbow extensions, with the surface EMG recorded from the long head of the triceps brachii muscle bellies. After the elbow extension MVIC test, the research staff placed the subjects’ wrist into a cuff, which was connected to one end of a force transducer (Model SM-500; Interface, Scottsdale, AZ, USA). With the force transducer connected to the immovable hook on the floor, the subjects were asked to flex their forearms. Extra care was taken to ensure the forearms were parallel to the floor. With this setup, the subjects performed three sets of 5-s MVICs with the hand supinated. At least 1-min rest was provided between both elbow flexors and extensors strength test sets. The highest 1-s portion of the MVICs was selected and calculated as the elbow flexor maximal isometric strength. For the maximal EMG amplitude for both biceps and triceps muscles, the highest 1-s EMG root-mean-square (RMS) window was selected during the maximal contractions, and then calculated as the maximal EMG amplitude, for the purpose of later EMG amplitude normalization.

**Figure 1:**
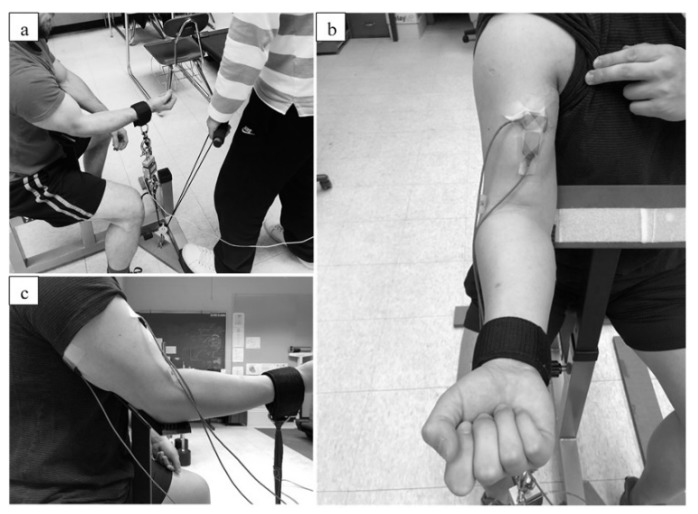
(**a**) The demonstration of the elbow flexion position task; (**b**) The electrodes’ location on the biceps muscle (the electrode on top is the 5-pin decomposition sensor); (**c**) The electrodes’ location on the triceps muscle.

At least two minutes after the maximal isometric tests, the subject performed submaximal trapezoid isometric elbow flexions with 40% and 70% of the pre-determined maximal isometric strength during that visit. During the Force-Task Visit, the subjects started elbow flexion isometrically and produced force gradually from 0% (rest) to 40% MVIC for 4 s (10% MVIC per second), held it for 10 s, and then gradually decreased the force output to 0% MVIC (relaxed state) for 4-s. The same force increasing/decreasing rate was used when the subject performed 70% MVIC trapezoid isometric contraction. A monitor was provided to the subjects to show the target force template and the real-time force. During the Position-Task Visit, a slightly different manner was used to conduct the trapezoid contractions. Instead of isometrically contracting against the force transducer, the subject was asked to maintain the forearm position (135-degree of elbow joint angle using 135° v-shaped steel hinge), while the research staff used a pulley system to gradually pull the other end of the force transducer, following the visual feedback on the monitor (Figure 1). Thus, visual feedback was not provided for the subjects. In addition, during this task, instructors provided verbal instructions and feedback to the subjects so they could maintain the designated elbow angle (135-degree with forearm at the horizontal position). If their forearm reached the v-shaped hinge during contractions, the investigators verbally notified them to make their forearm pulled back to the horizontal position. Prior to the onset of the position task contraction, the subject’s dominant hand was passively placed and held by the investigator, and the exact same trapezoid contractions were performed as they were during the Force-Task Visit. The 40% and 70% MVIC contractions were randomly sequenced, with two contractions performed for each contraction intensity. Between consecutive contractions, one 1-min rest period was provided. During these tasks, the subject was verbally encouraged to produce the force accurately or to maintain the 135-degree elbow joint angle.

### 2.4. Measurements

#### 2.4.1. Force

During all maximal and submaximal isometric elbow flexion contractions, the force was detected by the tension applied to the force transducer (Model SM-500; Interface, Scottsdale, AZ, USA). The signal was digitized with a 16-bit analog-to-digital converter (input range: 0–10 V, resolution = 0.153 mV; Model USB-6259; National Instruments, Austin, TX, USA). To compare the isometric force tracings during the submaximal trapezoid isometric contractions between the force and position tasks, we calculated the force error coefficient of variance (CoV) of each phase (force ramp-up; force plateau; force ramp-down) of the selected force signal (see the next section for the detailed selection criteria for the trapezoid submaximal isometric contraction). Specifcially, force error CoV = (standard deviation of the force error) ÷ (mean of the force error) × 100%. In addition, to assess the steadiness of the isometric force tracing, we also calculated the force CoV from the mid-6-s of the 10-s plateau region of each selected submaximal trapezoid isometric contraction, where force CoV = (standard deviation of the real-time force) ÷ (mean of the force) × 100%.

#### 2.4.2. Surface EMG Acquisition and Data Analyses

The EMG signals of biceps brachii were recorded using a specific decomposition surface EMG sensor (dEMG sensor, Delsys, Inc., Natick, MA, USA) consisting of 5 pins located on the corner and the center (placed on the muscle belly) (Figure 1b). Thus, this sensor generated 4 channels of differential bipolar EMG signals. In addition, bipolar surface electrodes (input impedance > 10^15^ Ω, DE 2.1 single differential surface EMG sensor, 10-mm interelectrode distance, Delsys, Inc., Natick, MA, USA) were also attached over the biceps brachii and the long head of the triceps brachii muscle bellies to record surface EMG signals based on the recommendations from SENIAM (Figure 1c) [25]. The reference electrode (Model USX2000; Axelgaard, Fallbrook, CA, USA) was then placed on the seventh cervical vertebrae (C7). Prior to any electrode placements, the investigator shaved and cleaned the skin surface with rubbing alcohol. The collected analog bipolar EMG signals were amplified (gain = 1000), and filtered with a bandpass filter of 20–450 Hz through a Bagnoli 16-channel EMG system (Delsys, Inc., Natick, MA, USA). The filtered signals were digitized at a sampling rate of 20,000 Hz with a 16-bit analog-to-digital converter (input range: 0–10 V, resolution = 0.153 mV; Model USB-6259; National Instruments, Austin, TX, USA). There was no saturation observed in the collected signals.

**Figure 2:**
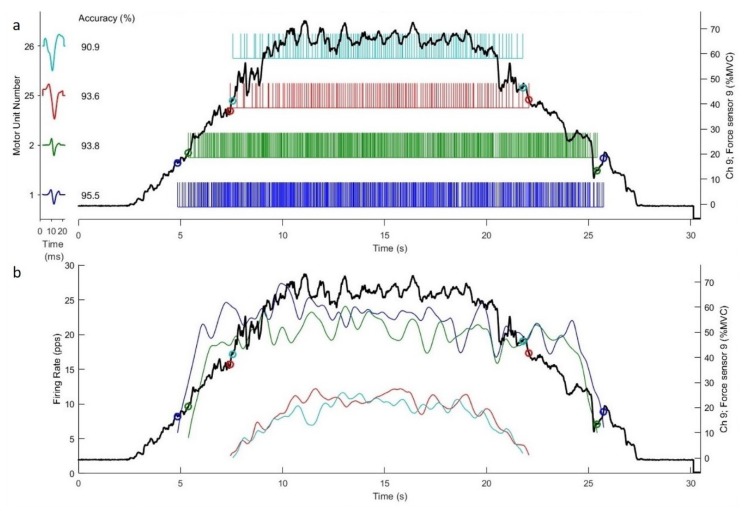
(**a**) Individual motor unit action potential trains during the isometric, trapezoid muscle action at 70% maximal voluntary isometric contraction (MVIC) intensity. Each vertical bar represents a motor unit firing. The solid black line is the subject’s force output. (**b**) Time-varying mean firing rate plots during the submaximal trapezoid isometric contraction. To enhance visual clarity, only the first two (blue and green circles) and last two (red and teal circles) recruited MUs have been displayed. Each colored line represents the mean firing rate (MFR) curve over time for an individual MU of the biceps brachii muscle. The mid-6-s of the trapezoid contraction is selected for MU MFR calculation.

After the acquisition of the surface EMG signals, the four separated EMG signals collected by the dEMG sensor were decomposed into constituent MU action potential trains using a software program (dEMG 1.1 Analysis, Delsys, Inc., Natick, MA, USA). This study included the MUs that could be decomposed with ≥ 90% accuracy, using the Decompose-Synthesize-Decompose-Compare (DSDC) test described by Nawab et al. [15]. The time-varying mean firing rate curve was obtained by convoluting the impulse trains corresponding to the firing trains of the MU through a 1-s Hanning window, using dEMG Analysis software (version 1.1, Delsys, Inc., Boston, MA, USA). To calculate the MFR of each MU, we selected the signals from the mid-6-s of the 10-s plateau region of each submaximal trapezoid isometric contraction. The MFR of each detected MU was then calculated. In addition, the RT and DT were defined as the first and last firing instances occurred of the decomposed MU, respectively, and expressed as the percentages of the maximal isometric strength (%MVIC) (Figure 2). Futhermore, examinations were conducted via the Decompose-Synthesize-Decompose-Compare (DSDC) test to ensure the first and last firing instances were true firing events of all the decomposed MUs. We then used linear regression analyses to explore the relationship between MU RT and MFR, as well as the relationship between MU RT and DT for each trapezoid isometric contraction [17]. Thus, each relationship yielded a linear slope coefficient and a y-intercept. The exclusion criterion included low coefficient of determination (r^2^ < 0.60). In addition, the trapezoid contraction that yielded a greater number of MUs and with a greater r^2^ for the linear regression analysis was selected for further statistical analysis. Lastly, for the selected 6-s plateau portion of the trapezoid contraction, the RMS of the EMG signals for both biceps and triceps muscles were calculated. All EMG RMS values were normalized as the percentages of the maximal EMG values from the isometric strength tests.

### 2.5. Statistical Analyses

First, assumption of dependent variables and regression were tested. A Shapiro–Wilk test supported that distributions of dependent variables were normal, and both Skewness and Kurtosis values were within range of ±2. Histogram and Normal P–P plots for regression indicated that distributions of standardized residuals were normal. Paired samples *t*-test was used to compare the maximal elbow flexion isometric strength values and the range of recruitment threshold of recorded MUs between the two experimental visits. To compare the isometric force tracings between two tasks, a three-way (condition [force vs. position] × force tracing phase [ramp-up vs. plateau vs. ramp-down] × contraction intensity [40% vs. 70%]) repeated measures analysis of variance test (ANOVA) was conducted to compare force error CoV between two tasks. Lastly, the force steadiness during the mid-6-s plateau region, the biceps and triceps EMG amplitude, and the biceps muscle MU firing properties (linear slope coefficients and y-intercepts of the MU MFR vs. RT as well as the RT versus DT relationships) during different isometric tasks at different contraction intensities were examined via separate two-way (condition [force vs. position] × intensity [40% vs. 70%]) repeated measures ANOVAs. The partial $\eta^{2}$ statistics are provided for all repeated measure comparisons, with values of 0.01, 0.06, and 0.14 corresponding to small, medium, and large effect sizes, respectively [26]. In addition, Cohen’s *d* was also calculated for paired comparisons, with 0.2, 0.5, and 0.8 corresponding to small, medium, and large effect sizes, respectively, when necessary [26]. Dependent variables were reported as mean ± SD unless stated otherwise. All statistical tests were conducted using statistical software (IBM SPSS Statistics 22.0, IBM, Armonk, NY, USA) with alpha set at 0.05.

## 3. Results

### 3.1. Test–Retest Reliability

The intraclass correlation coefficient model (3, 1) (ICC_3,1_) [27] for the elbow flexors’ maximal isometric strength during three visits (familiarization visit vs. force-task visit vs. position-task visit) was reliable (r = 0.97), with no significant difference among the three visits (F = 0.25, *p* = 0.782).

### 3.2. Isometric Force and EMG Amplitude during Submaximal Isometric Trapezoid Contractions

The paired sample *t-*test indicated no significant difference for maximal isometric strength between two experimental visits (force-task visit vs. position-task visit = 299.3 ± 133.7 N vs. 299.4 ± 132.4 N, *p* = 0.987, *d* = 0.001). For the force error CoV, the 3-way ANOVA only showed a 2-way phase × intensity interaction (F = 5.45, *p* = 0.032, partial$\;\eta^{2}$ = 0.280), and a main effect for phase (F = 78.176, *p* < 0.001, partial$\;\eta^{2}$ = 0.848). In addition, based on the result of the 2-way ANOVA, the force steadiness (force CoVs) during the mid-6-s of the plateau regions of the two isometric tasks were also not significantly different (F = 2.79, *p* = 0.117, partial$\;\eta^{2}$ = 0.166).

For the biceps EMG amplitude, the results from the 2-way ANOVA showed no interaction (F = 0.25, *p* = 0.621, partial$\;\eta^{2}$ = 0.014) or main effect for condition (F = 1.43, *p* = 0.248, partial$\;\eta^{2}$ = 0.073), but there was a main effect for intensity (F = 75.22, *p* < 0.001, partial$\;\eta^{2}$ = 0.807), indicating greater EMG amplitude during 70% MVIC, when compared to 40% MVIC (tasks merged, 40% MVIC vs. 70% MVIC = 57.5 ± 19.8% vs. 105.1 ± 30.2%, *p* < 0.0001, *d* = 1.86). For the normalized triceps brachii EMG amplitude, the 2-way ANOVA showed no interaction (F = 1.19, *p* = 0.290, partial$\;\eta^{2}$ = 0.062) or main effect for condition (F = 0.22, *p* = 0.644, partial$\;\eta^{2}$ = 0.012), but there was a main effect for intensity (F = 31.94, *p* < 0.001, partial$\;\eta^{2}$ = 0.640). After collapsing the across condition, 70% MVIC contraction intensity generally showed a greater EMG amplitude (both tasks merged) than 40% MVIC (40% MVIC vs. 70% MVIC = 12.4 ± 8.6% vs. 23.5 ± 16.6%, *p* < 0.0001, *d* = 1.88).

### 3.3. Motor Unit Firing Properties

**Figure 3:**
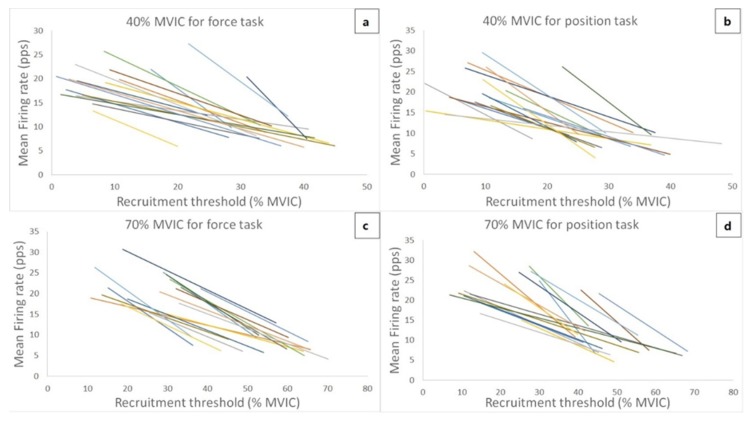
The theoretical regression lines obtained from the recruitment threshold (RT) versus the mean firing rate (MFR) relationship for 17 subjects during the force task and position task at 40% and 70% maximal voluntary isometric contraction (MVIC).

Figure 3 shows the theoretical regression lines for the relationship between the MU RT and MFR for all 17 subjects during 40% and 70% MVIC force task and position task isometric contraction. Due to the low-quality data and exclusion criterion, two subjects were excluded for linear regression analysis. Thus, the linear regression for the relationship between MU RT and MFR was analyzed from seventeen subjects. The ranges of recruitment thresholds of recorded MUs were similar between the two different tasks at 40% (force task vs. position task = 8.37 ± 7.92% to 34.91 ± 6.86% vs. 8.70 ± 5.51% to 31.63 ± 7.45%) and 70% MVIC contraction intensities (force task vs. position task = 24.41 ± 8.48% to 55.25 ± 10.47% vs. 20.40 ± 11.26% to 50.51 ± 9.56%). For the linear slope coefficients, the 2-way ANOVA showed no significant interaction (F = 0.05, *p* = 0.826, partial$\;\eta^{2}$ = 0.003) or main effect for intensity (F = 1.71, *p* = 0.209, partial$\;\eta^{2}$ = 0.097), but there was a significant main effect for condition (F = 6.73, *p* = 0.020, partial$\;\eta^{2}$ = 0.296). The follow-up pairwise comparison indicated that the slope coefficient was significantly more negative (intensity merged) during the position task, when compared to the force task (force task vs. position task = -0.48 ± 0.24 pps/%MVIC vs. −0.63 ± 0.27 pps/%MVIC, *p* = 0.010, *d* = 0.588). For the y-intercepts, the 2-way ANOVA did not show a significant interaction (F = 1.08, *p* = 0.314, partial$\;\eta^{2}$ = 0.063) or main effect for condition (F = 1.90, *p* = 0.187, partial$\;\eta^{2}$ = 0.106). However, there was a significant main effect for intensity (F = 13.56, *p* = 0.002, partial$\;\eta^{2}$ = 0.459), indicating that the y-intercepts (condition merged) were higher during the 70% than 40% MVIC contraction intensity (40% MVIC vs. 70% MVIC = 26.75 ± 10.83 pps vs. 34.75 ± 11.03 pps, *p* < 0.001, *d* = 0.734).

**Figure 4:**
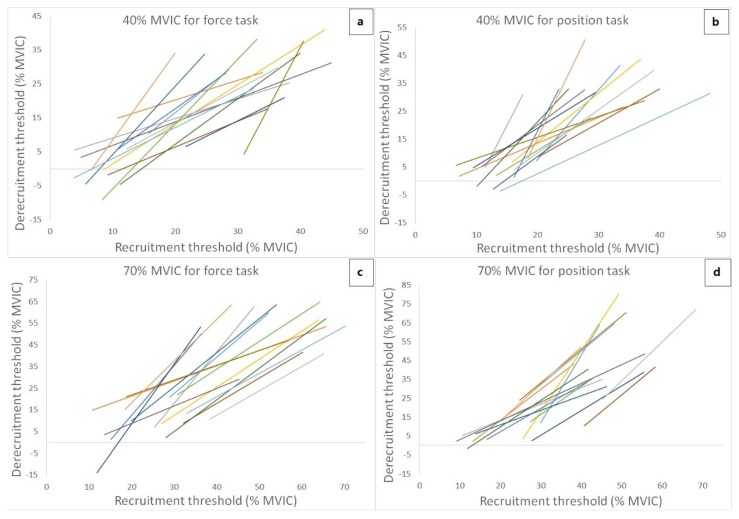
The theoretical regression lines obtained from the recruitment threshold (RT) versus the derecruitment threshold (DT) relationship for 15 subjects during the force task and position task at 40% and 70% maximal voluntary isometric contraction (MVIC).

Figure 4 shows the theoretical regression lines for the relationship between the MU RT and DT for each of 15 subjects during 40% and 70% MVIC force task and position task isometric contraction. Four of the subjects were excluded for linear regression of 40% and 70% MVIC of trapezoid contracions due to MU data exclusion criteria. Thus, fifteen subjects were collected MU data for 40% and 70% MVIC trapezoid contractions. The number of MUs recorded, the number of MUS with RT < DT and RT > DT, and cross-over point (RT MVIC%) from the relationship between the MU RT and DT during both isometric tasks are presented in Table 1 (40% MVIC contraction intensity) and Table 2 (70% MVCI contraction intensity) for each subject. The cross-over point for force task and position task at 40% MVIC were varied with an average of 23.56% ± 6.97% and 19.60% ± 3.54%, respectively. In addition, the cross-over point varied with an average of 41.14% ± 14.34% and 39.25% ± 9.82% for force task and position task at 70% MVIC, respectively. The results from the 2-way ANOVA showed that there was no significant interaction (F = 0.34, *p* = 0.571, partial$\;\eta^{2}$ = 0.024) or main effect for intensity (F = 1.04, *p* = 0.325, partial$\;\eta^{2}$ = 0.069), but there was a significant main effect for condition (F = 4.81, *p* = 0.046, partial$\;\eta^{2}$ = 0.256) for the linear slope coefficient. The follow-up pairwise comparison indicated that the slope coefficient was significantly more positive (intensity merged) during the position task, when compared to the force task (force task vs. position task = 1.36 ± 0.67 pps/%MVIC vs. 1.70 ± 0.74 pps/%MVIC, *p* = 0.023, d = 0.486). For the y-intercepts, the 2-way ANOVA showed that there was no significant interaction (F = 0.78, *p* = 0.392, partial$\;\eta^{2}$ = 0.053) or main effect for condition (F = 1.88, *p* = 0.192, partial$\;\eta^{2}$ = 0.118). However, there was a significant main effect for intensity (F = 5.16, *p* = 0.039, partial$\;\eta^{2}$ = 0.269), indicating that the y-intercepts (conditions merged) were lower during the 70% than 40% MVIC contraction intensity (40% MVIC vs. 70% MVIC = −15.45 ± 20.02 pps vs. −29.24 ± 23.45 pps, *p* = 0.020, *d* = 0.632).

## 4. Discussion

The main purpose of this study was to examine MU control strategies between a force and position task for the elbow flexors. The linear regression analysis was specifically used to examine the relationship between the MU RT and MFR, as well as the relationship between MU RT and DT at two contraction intensities (40% and 70% MVICs). In addition, global surface EMG amplitude was also examined for both agonist (biceps brachii) and antagonist (triceps brachii) muscles. The main findings of this study were: (1) The surface EMG amplitude did not differ between two tasks at both intensities for both agonist and antagonist muscles; and (2) The linear slope coefficients of the MU RT vs. MFR as well as the RT vs. DT relationships were significantly different between the two tasks (contraction intensities merged).

The elbow flexion isometric strength values were not different between the two experimental visits. The results of the agonist and antagonist EMG amplitude responses during the two tasks were generally in agreement with previous studies [10,11] that compared the EMG amplitude between these tasks. For example, Garner et al. [10] reported no difference in the soleus muscle EMG amplitude between two tasks during non-fatiguing plantarflexions. In addition, Buchanan and Lloyd [11] examined the EMG amplitude of biceps brachii, brachialis, brachioradialis, and triceps brachii during force and position elbow flexion tasks with relatively low loads (up to 40% of the maximal strength). It was reported that there was large individual variation (six out of nine individuals showed greater activation level during position task than force task) for the biceps brachii EMG amplitude between the two tasks, and no difference in antagonist muscle (triceps) EMG amplitude was shown [11]. It is worth mentioning that in the current study, the condition factor imposed a medium effect on the biceps EMG responses (partial$\;\eta^{2}$ = 0.073): 14 out of 19 and 13 out of 19 participants demonstrated greater EMG amplitude during 40% and 70% MVIC contraction intensities during position tasks, respectively, when compared to the force tasks. In addition, there were differences in MU firing properties (the relationship between RT vs. MFR and RT vs. DT) between the two tasks despite no significant difference in EMG amplitude.

Our results of MU firing properties show an interesting finding: the slope coefficients of the MU RT vs. MFR and RT vs. DT relationships were more negative and more positive, respectively, during the position task when compared to these during the force task (both intensities merged). According to De Luca and Hostage [17], the linear relationship between the MU RT and MFR describes an “operating point” of the motoneuron pool responding to different levels of excitation. With a greater level of contraction intensity (force output), the slope and y-intercept of this relationship become less negative and greater, respectively, suggesting the recruitment of the high-threshold MUs, as well as the increased firing rates of the active MUs. The current finding showed a more negative slope coefficient for the MU RT vs. MFR relationship under the position task, as compares to the force task (contraction intensities merged). This, therefore, confirms the different MU control strategies used between these tasks. It seems that compared to the force task contraction, the recruitment of the relatively high-threshold MUs might not be the main strategy to reach the same force output when performing the position task contraction. For the relationship between the MU RT and DT, it has been shown to be linear and positive in previous studies. [17,18]. Generally speaking, low-threshold and high-threshold MUs are usually derecruited at lower force and at higher force levels, respectively, than which they were recruited (De Luca and Hostage 2010; De Luca et al. 1982; Farina et al. 2009; Stock and Mota 2017), indicating that with the same absolute high force output, fewer active MUs are needed during derecruitment than during recruitment. This, however, may not always be true, because different muscles can add variation to this relationship [17,28]. In the current study, the more positive slope coefficient of the MU RT vs. DT relationship was found during the position task than that during the force task. This suggests that based on the theoretical slope regression line shown in the results, active MUs during the position task derecruited at higher force levels than where they were recruited. It also indicates that, when compared to the same absolute force levels between two isometric tasks, fewer MUs were required during derecruitment (force reducing phase) in the position task than that in the force task.

Our results of the y-intercepts of MU RT vs. MFR and RT vs. DT relationships were greater and lower at 70% MVIC than at 40% MVIC, respectively (both tasks merged). The increase of the force output from 40% to 70% MVIC is largely due to the greater neural drive to the motoneuron pools of the active muscles, inducing the recruitment of additional high-threshold MU, the increased firing rates of the active MUs, and the declined derecruitment threshold [12,17]. Thus, considering the y-intercepts of the MU RT vs. MFR and RT vs. DT relationships indicate the theoretical maximal firing rates and derecruitment of the active MU [17], the results of the y-intercepts were within our expectation and in agreement with previous studies [12,17].

The current study found the task-related differences in the slopes of the linear relationships between the MU RT and MFR, and between the RT and DT during the non-fatiguing contractions. However, it is unclear what mechanism(s) could have contributed to these differences. There are several worth-mentioning limitations for this experiment. First, although the reflex-related mechanism may support the current findings, it is still speculative to link the current MU control results to this mechanism without any caution, because we were not able to examine the reflex responses for both tasks. Second, methodological-related limitations should also be identified to improve similar future experiments. For example, EMG was not normalized to the maximal compound muscle action potential or collected from synergist muscles. In addition, the execution of the submaximal trapezoid contraction during the position task could also be influenced by the force production from the research staff.

## 5. Conclusions

In conclusion, even though there were no differences in agonist and antagonist EMG amplitude responses between two different isometric tasks, the present study found task-related differences in MU control strategies between the force and position tasks. The recruitment of high-threshold MUs and the number of active MUs at derecruitment could have contributed to the task-related difference between two tasks. To reach and maintain the same force output, the position task may rely less on the recruitment of relatively high-threshold MUs, as compared to the force task. In addition, as the force output decreases, MUs tend to derecruit at higher force levels during the position task than during the force task. However, the exact mechanisms for the different MU control strategies between two tasks remain unclear. To further understand the underlying mechanism(s) for MU control strategies for the two isometric tasks, it is necessary to examine the changes of excitatory and inhibitory input that occur in the spinal or supraspinal level. The changes in heteronymous H-reflex can estimate modulation monosynaptic input to the spinal cord and is likely mediated by presynaptic inhibition of Ia afferent to the muscle. Thus, the potential difference in reflex responses, as well as the difference in MU activities between isometric tasks could be explored in future research.

Isometric muscle performance is a common measurement to evaluate neuromuscular functions and fitness levels in the field of sports science [29]. In addition, isometric muscle action is an important means for patients during the rehabilitation process. Despite similar mechanical requirements for both isometric tasks, the current results suggest that force and position tasks seem to utilize different MU control strategies. This task-related difference can provide important information when designing experiments in the field of sports science, or designing exercise programs for patients in rehabilitation settings. Implementing different isometric tasks during the rehabilitation may yield distinct benefits for certain clinical populations.

## Figures and Tables

**Table 1:** The number of motor units, the number of motor units with recruitment threshold (RT) < derecruitment threshold (DT) and RT > DT, and cross-over point (RT%) of the motor unit RT and DT relationship for force and position tasks at 40% maximal voluntary isometric contraction (MVIC) contraction intensities.

40% MVIC		RT vs. DT					RT vs. DT			
	Subject	# MUs	#RT > DT	#RT < DT	Cross-Over Point (RT%)		# MUs	#RT > DT	#RT < DT	Cross-Over Point (RT%)
Force task	1	15	12	3	23.92, 27.40†	Position task	13	10	3	26.33
	2	9	2	7	33.77*		24	24	0	-
	3	25	20	5	13.65*		21	8	13	12.76
	4	38	31	7	21.08, 28.17†		26	16	10	24.19
	5	19	17	2	24.68		16	12	4	19.96
	6	29	13	16	28.64		15	15	0	-
	7	15	8	7	19.39		20	20	0	-
	8	22	22	0	-		25	25	0	-
	9	24	22	2	5.60, 9.40†		20	14	7	21.00
	10	23	20	3	39.78, 40.50†		16	15	1	11.92*
	11	22	22	0	-		10	9	1	25.38, 27.71†, 29.50†
	12	23	23	0	-		24	12	12	18.23
	13	22	21	1	32.46		24	24	0	-
	14	9	2	7	12.63		26	15	11	21.44
	15	17	17	0	-		20	19	1	37.91, 39.00†
	Mean	20	-	-	23.56‡		20	-	-	19.60‡‡
	SD	7	-	-	6.97		5	-	-	3.54

* indicated low-threshold motor unit derecruited at higher force levels than where they were recruited; † indicated reversal between RT and DT relationship; ‡ include only subject 5, 6, 7, 13, and 14 for the force task; ‡‡ include only subject 3, 4, 5, 9, 12, and 14 for the position task.

**Table 2:** The number of motor units, the number of motor units with recruitment threshold (RT) < derecruitment threshold (DT) and RT > DT, and cross-over point (RT%) of the motor unit RT and DT relationship for force and position tasks at 70% maximal voluntary isometric contraction (MVIC) contraction intensities.

70% MVIC		RT vs. DT					RT vs. DT			
	Subject	# MUs	#RT > DT	#RT < DT	Cross-Over Point (RT%)		# MUs	#RT > DT	#RT < DT	Cross-Over Point (RT%)
Force task	1	23	17	5	46.34	Position task	25	15	10	36.02
	2	19	17	2	26.38*		22	12	9	34.34
	3	17	8	9	39.35		27	27	0	-
	4	27	27	0	-		31	12	19	38.59
	5	23	11	12	24.93		28	28	0	-
	6	19	14	5	43.26, 48.10†, 48.62†, 57.90†		19	19	0	-
	7	20	11	9	31.38, 36.68†, 38.42†		19	19	0	-
	8	19	19	0	-		18	18	0	-
	9	24	23	1	42.94, 45.22†		31	26	5	39.44, 44.65†
	10	21	18	3	37.11*		27	6	21	25.79, 31.53†, 39.59†,
	11	20	12	8	29.46		20	20	0	-
	12	16	15	1	65.61		22	19	3	40.81
	13	21	21	0	-		25	6	19	26.83
	14	25	0	25	-		17	5	12	30.56*, 34.18†,
	15	14	14	0	-		29	22	7	58.92
	Mean	21	-	-	41.14‡		24	-	-	39.25‡‡
	SD	3	-	-	14.34		5	-	-	9.82

* indicated low-threshold motor unit derecruited at higher force levels than where they were recruited; † indicated reversal between RT and DT relationship; ‡ include only subject 1, 3, 5, 11, and 12 for the force task; ‡‡ include only subject 1, 2, 4, 12, 13, and 15 for the position task.
